# Gut-derived butyrate suppresses ocular surface inflammation

**DOI:** 10.1038/s41598-022-08442-3

**Published:** 2022-03-16

**Authors:** Laura Schaefer, Humberto Hernandez, Rosalind A. Coats, Zhiyuan Yu, Stephen C. Pflugfelder, Robert A. Britton, Cintia S. de Paiva

**Affiliations:** 1 Center of Metagenomics and Microbiome Research, Department of Molecular Virology and Microbiology, Baylor College of Medicine, 1 Baylor Plaza, Houston, TX 77030 USA; 2 Ocular Surface Center, Department of Ophthalmology, Cullen Eye Institute, Baylor College of Medicine, 6565 Fannin St., NC505, Houston, TX 77030 USA; 3 Present Address: Humberto Hernandez, University of Houston-Victoria, 3007 N. Ben Wilson, Victoria, TX 77901 USA

**Keywords:** Corneal diseases, Microbiome, Conjunctival diseases

## Abstract

Dry eye is a common ocular inflammatory disorder characterized by tear film instability and reduced tear production. There is increasing evidence that homeostasis of the ocular surface is impacted by the intestinal microbiome. We are interested in investigating the potential role of microbially produced small molecules in mediating the interaction between the intestinal microbiota and the ocular surface. One such molecule is butyrate, a short-chain fatty acid (SCFA) produced by certain members of the gut microbiota through fermentation of dietary fiber. Here we show that SCFA transporter SLC5A8 is expressed in vivo in murine conjunctival and corneal epithelium. Pre-treatment of in vitro corneal epithelial cultures or bone marrow-derived dendritic cells (BMDCs) with phenylbutyrate (PBA) reduces lipopolysaccharide-induced pro-inflammatory *Tnf* expression. Corneal epithelial cultures and BMDCs isolated from *Slc5a8* knockout mice are unable to respond to PBA pre-treatment, suggesting that SLC5A8 is required for the protective effect of PBA. The treatment of mice undergoing desiccating stress (DS) with oral tributyrin, a prodrug form of butyrate, reduces inflammation at the ocular surface in vivo*,* and this effect partially requires SLC5A8. Finally, expression analysis on conjunctival tissue isolated from mice subjected to DS with and without tributyrin treatment revealed that treatment downregulated genes involved in Type I interferon signaling. Together these data support our hypothesis that SCFAs produced in the gut participate in the maintenance of ocular surface homeostasis.

## Introduction

Dry eye is a chronic multifactorial condition in which the eyes do not produce enough tears or have a disruption in tear film stability. Major risk factors include aging, female gender, contact lens use, smoking, and low humidity environments^1^. The unstable and hyperosmolar tear film in dry eye triggers a self-perpetuating inflammatory cascade involving innate and adaptive immune cells and causes eye irritation, hyperemia, eye fatigue, and blurred vision^2,3^. Inflammation at the ocular surface results in goblet cell apoptosis in the conjunctival epithelium, corneal epithelial barrier disruption, and lacrimal gland inflammation and dysfunction^2^. In severe cases, this can cause sight threatening corneal epithelial disease and ulceration.

The commensal bacteria that inhabit the gastrointestinal tract have far-reaching effects on health and homeostasis in the intestine and at distal body sites, including the brain, bone, and barrier organs like lung, kidney, and eye^4–10^. One important function mediated by gut microbiota is the maintenance of immune homeostasis at mucosal barrier tissues throughout the body. The ocular surface represents one such barrier tissue; similar to the intestinal luminal barrier, the cornea is constantly exposed to foreign stimuli such as environmental stressors and microbial pathogens, and under healthy normal conditions maintains an equilibrium between immune tolerance and immunity^2^. The gut microbiota influences inflammatory responses by modulating immune cell maturation and function, including ﻿promoting the production of regulatory T cells (Tregs) and tolerogenic dendritic cells^11–13^. The lack of commensal bacteria in germ-free mice results in underdeveloped lymphoid tissue, deficient production of the secretory antibody IgA (SIgA) and antimicrobial peptides, and abnormal cytokine production^14–17^. In fact, the presence of gut commensals has been shown to modulate SIgA levels in tears and in eye-associated lymphoid tissue, as germ-free and antibiotic-treated mice show a significant decrease in ocular SIgA and mice reconstituted with certain commensals show restoration of SIgA^18,19^. Healthy normal gut microbiota is also protective against the development of inflammatory dry eye pathology in a murine desiccating stress model^20^. This is supported by the observation that germ-free mice, which are raised in the absence of bacteria, spontaneously develop lacrimal keratoconjunctivitis; fecal microbiota transplant reverses the dry eye phenotype in germ-free mice^21^. Likewise, ocular disease severity in CD25 knockout mice, which also spontaneously develop dry eye disease, is worsened by disruption of the microbiota, either with antibiotic treatment or by raising the mice in a germ-free environment, and is improved by subsequent fecal microbiota transplant^22^.

One mechanism by which commensal bacteria may impact their host is through the secretion of factors that can interact with host cell signaling pathways or modulate host gene expression. Short-chain fatty acids (SCFAs), including butyrate, propionate, and acetate, are metabolites that are produced in the gut by the commensal microbiota from the fermentation of dietary fiber. SCFAs have been shown to exert anti-inflammatory effects that extend beyond the colon into other systems, including impacts on both adaptive and innate immune cells, bone homeostasis, the brain, and the eye^23–29^. Clear links between SCFAs in the gut and ocular health have been demonstrated in models for uveitis^26,27^. In addition to exerting effects indirectly through the adaptive and innate immune systems, butyrate may also act directly on ocular tissue. Direct application of butyrate to the eye has been demonstrated to improve wound healing and corneal opacification of alkali burns through inhibition of the NLRP3 inflammasome^30^. However, the effects of butyrate during desiccating stress has not been fully investigated. In this study, we examined the potential role of butyrate in mediating the interaction between the gut microbiota and the ocular surface.

## Materials and methods

### Animals

Female wild-type mice (C57BL/6 J strain) were purchased at 6 to 8 weeks of age from Jackson Laboratory (Bar Harbor, ME, USA). *Slc5a8* knockout mice were a gift from Dr. Vadivel Ganapathy (Texas Tech University, Lubbock, Texas) and were originally derived by Dr. Thomas Boettger (Max Planck Institute, Germany)^31^. Animal studies were approved by the Institutional Animal Care and Use Committee at the Baylor College of Medicine and adhered to the Association for Research in Vision and Ophthalmology Statement for Use of Animals in Ophthalmic and Visual Research. A total of one hundred female wild-type mice and fifty female *Slc5a8* knockout mice were used in the course of the study. The number of mice per experiment is given in the sections below and also in figure legends. At the time of sacrifice, mice were 8 to 12 weeks old. All studies are reported in accordance with the ARRIVE guidelines^32^.

### Digital PCR

Murine corneal epithelium and conjunctival tissue were collected from wild-type C57BL/6 J mice according to previously published methods^20^. In brief, total RNA was extracted with Qiagen RNeasy Plus Micro RNA isolation kit (Qiagen, Germantown, MD, USA) following the manufacturer’s protocol. One sample equaled the tissue pooled from both eyes of each animal. cDNA was synthesized using Ready-To-Go First-Strand beads (GE Healthcare Life Sciences, Marlborough, MA, USA) and random hexamers (Life Technologies, Grand Island, New York, USA) with 1 µg total RNA as template. DNA concentration was measured with a Qubit spectrophotometer (Life Technologies). Digital polymerase chain reaction (PCR) was performed as previously described with a QuantStudio 3D Digital PCR system (Life Technologies) with *Slc5a8* Taqman assay primer set Mm00520629_m1 (Applied Biosystems, Inc. [ABI], Foster City, CA) and normalized by concentration of cDNA^33^.

### Histology and Immunostaining

For evaluation of morphology, eyes and ocular adnexa were excised, embedded in paraffin wax, and cut into 8-μm sections. Sections were stained with hematoxylin and eosin (H&E). For expression analysis, eyes and ocular adnexa were excised, embedded in Tissue-Tek Optimal Cutting Temperature medium (OCT; Sakura Finetek, Torrance, CA, USA) blocks and flash-frozen in liquid nitrogen, then cut into 6-μm sections using an HM 500 cryostat (Waldorf, Germany). Tissue sections of at least 3 different animals were fixed in 4% paraformaldehyde, and protein expression was visualized with chromogenic staining with a rabbit polyclonal antibody to SLC5A8 (Proteintech #21,433–1-AP, Rosemont, IL, USA) according to previously published methods^34^. Separate sections were incubated with rabbit IgG (Sigma-Aldrich, St. Louis, MO, USA) as a negative control for primary antibody specificity. Antibodies were diluted according to manufacturer recommendations in 5% goat serum (Sigma-Aldrich) in phosphate-buffered saline (PBS). Sections were prepared for immunohistochemistry using an avidin/biotin blocking kit (Vector Laboratories, Burlingame, CA, USA) after quenching endogenous peroxidases with 0.3% hydrogen peroxide and washing with PBS. Sections were blocked for 1 h with 20% goat serum in PBS and subsequently incubated with diluted primary antibody for 1 h. ﻿Pictures were taken at 60X magnification for H&E-stained sections and 40X magnification for immunostained sections with an E400 microscope equipped with a digital camera (DS-Qi1Mc; Nikon Instruments Inc, Melville, NY, USA).

### Cornea tissue explant cultures

Murine corneas were excised under a surgical microscope using curved Castroviejo scissors into a supplemented hormonal epidermal medium (SHEM) on ice containing 5 µg/ml dispase (Sigma-Aldrich). SHEM media was prepared as a 1:1 media mixture of Dulbecco’s Modified Eagle’s Medium with high glucose (Sigma-Aldrich) and Ham’s F-12 media (﻿Sigma-Aldrich) containing 5 ng/ml EGF (Thermo Fisher Scientific, Waltham, MA, USA), 0.5 mg/ml hydrocortisone (﻿Sigma-Aldrich), 30 ng/ml cholera toxin A (﻿Sigma-Aldrich), 0.5% DMSO (﻿Sigma-Aldrich), 50 mg/ml gentamicin (Hyclone, GE Healthcare Life Sciences, Marlborough, MA, USA), 1.25 mg/ml amphotericin B (﻿Gibco BRL Products, Grand Island, NY, USA), 1X ITS liquid media supplement (Sigma-Aldrich) and 5% FBS (Hyclone). The corneas were cut into four equal pieces, incubated at 37 °C for 15 min, and transferred with sterile forceps into SHEM media without dispase. After approximately 10 min, ﻿the pieces were transferred into wells of a 48-well culture plate with their epithelium side up, allowed to adhere for several minutes, then covered with 200 µl SHEM media and incubated at 37C in 5% CO_2_. 200 µl fresh SHEM media was added every 3 days. On day 13, the media was replaced with 300 µl SHEM media lacking FBS. On day 14, half the wells were treated with 5 µl 5 mg/ml phenylbutyrate (PBA; Sigma-Aldrich) prepared in 1:1 Dulbecco’s Modified Eagle’s Medium with high glucose (Sigma-Aldrich) and Ham’s F-12 media (Sigma-Aldrich) for a final concentration of 0.5 mM. The other wells were treated with 1:1 media without PBA. After 2 h, a subset of wells was treated with 0.3 µg lipopolysaccharide (LPS; Sigma-Aldrich) in 1:1 media, and the plate incubated for 4 h at 37C in 5% CO_2_ before adherent cells were collected for RNA isolation. Three separate experiments were performed using two to four each wild-type and *Slc5a8* knockout mice. In an initial pilot experiment, we tested 0.5 mM, 1 mM and 5 mM PBA on corneal cultures in order to identify an effective dose. There was no difference observed in the ability of these three concentrations to reduce the inflammation response. Based on this, we chose to use the lowest (0.5 mM) concentration in subsequent in vitro experiments.

### Bone marrow-derived dendritic cell (BMDC) cultures

Bone marrow cells were obtained by flushing cells from femurs of female C57BL/6 J or *Slc5a8* knockout mice with complete RPMI 1640 media (Gibco BRL) containing 10% FBS, 50 μg/ml gentamicin, and 1.25 μg/ml amphotericin B. Cells were filtered through a Sysmex CellTrics cell strainer (Sysmex, Lincolnshire, IL, USA). Marrow cells were cultured for 6 days at 10^7^ cells per well in 10-cm-diameter plates in complete RPMI 1640 medium supplemented with mouse granulocyte–macrophage colony-stimulating factor (GM-CSF; 20 ng/ml) and IL-4 (5 ng/ml; PeproTech, Rocky Hill, NJ, USA). On day 3, a fresh medium containing GM-CSF and IL-4 was added. On day 6, cells were collected from the plates and seeded into a 24-well plate with 10^6^ cells/well in 1 ml complete RPMI containing GM-CSF and IL-4. At this time a subset of wells received 18 µl 5 mg/ml PBA for a final concentration of 0.5 mM. On day 8, appropriate wells were treated with 1 µg/ml LPS for 4 h. Cells were harvested, centrifuged, and resuspended in Qiagen RLT buffer (Qiagen, Germantown, MD, USA) containing beta-mercaptoethanol (Sigma-Aldrich) buffer for RNA isolation. Two wells were combined for each sample. Each experiment used two to three each wild-type and *Slc5a8* knockout mice. Three separate experiments were performed.

### RNA Isolation and Real-Time Quantitative PCR

Cultured corneal epithelium cells and BMDCs were eluted in Qiagen RLT buffer containing beta-mercaptoethanol (Sigma-Aldrich) using 350 µl per well, and RNA was extracted using Qiagen RNeasy Plus (Qiagen, Germantown, MD, USA) according to the manufacturer protocol. The concentration of isolated RNA was measured using a Nanodrop 2000 spectrophotometer (Thermo Fisher Scientific). After RNA isolation, cDNA was synthesized using Ready-To-Go You-Prime First-Strand beads (GE Healthcare Life Sciences, Marlborough, MA, USA).

Conjunctival tissue collected from female C57BL/6 J mice was placed in Qiagen RLT buffer with beta-mercaptoethanol and flash-frozen in liquid nitrogen. For RNA isolation, tissue was chopped with surgical scissors then sonicated, and RNA was extracted using Qiagen RNeasy Plus.

Real-time PCR was performed by using specific TaqMan assays (TNF-α: ABI assay ID Mm00443260_g1; HPRT: Assay ID Mm00446968_m1; Slc5a8: Mm07300153_m1) with TaqMan CR Universal PCR Master Mix with AmpErase UNG; (Applied Biosystems), in a ﻿QuantStudio 3 real-time PCR system (ABI) according to the manufacturer’s recommendations. The results of quantitative PCR were analyzed by the comparative threshold cycle (C_T_) method and normalized by the C_T_ of HPRT. The relative mRNA level in the LPS-only treated group was used as the calibrator for each experiment. The data encompass the results of three separate experiments.

### Desiccating stress and tributyrin treatment

Mice were exposed to desiccating stress (DS) conditions a drafty low humidity (< 30% relative humidity) environment according to the standard desiccating stress methods previously described^20^, which included QID pharmacological inhibition of tear secretion by subcutaneous injection of scopolamine hydrobromide (0.5 mg/ 0.2 mL; Sigma-Aldrich, St. Louis, MO, USA). ﻿Mice were euthanized after 5 continuous days of DS.

Mice received daily gavage of 100 μl of 0.5 mM of tributyrin (W222305, Sigma-Aldrich) or of PBS (Corning, Manassas, VA, USA) once daily while subjected to desiccating stress. This dose was chosen after a pilot study that evaluated the effects on the corneal barrier after oral gavage of different concentrations of tributyrin (0.1 mM, 0.5 mM, 1 mM, and 5 mM in non-stressed mice, 0.5 mM and 5 mM in DS mice). In non-stressed mice, all doses were not significantly different from untreated controls, indicating there was no toxicity or deleterious effect to the ocular surface. In DS mice, since the lower dose was efficacious, we performed subsequent experiments with 0.5 mM tributyrin (data not shown).

### Measurement of corneal barrier function

Corneal barrier function was assessed by quantifying corneal epithelial permeability to 70-kDa Oregon Green Dextran-488 (OGD; Invitrogen, Carlsbad, CA) according to our previously published protocol^22^. Each image was quantified by two blinded observers. The mean intensity of the right and left eyes was averaged. Initial experiments with wild-type mice utilized nine to fifteen (18–30 eyes) mice per experimental group. Subsequent experiments with both wild-type and *Slc5a8* knockout mice used eight to ten mice (16–20 eyes) per experimental group. ﻿

### Measurement of goblet cell density

Eyes and ocular adnexa were excised, fixed in 10% formalin, embedded in paraffin, and cut into 5-μm sections using a microtome (Microm HM 340E, Thermo Fisher Scientific). Sections were stained with Periodic Acid Schiff (PAS) reagent to visualize the mucin-containing goblet cells. The goblet cell density was measured in the superior and inferior conjunctiva using NIS-Elements software and expressed as the number of positive cells per millimeter^22^. Five to nine eyes per each group were examined. Each image was quantified by two blinded observers.

### Statistical analysis

Statistical analysis was performed with GraphPad Prism 9.0 software (GraphPad Software Inc., San Diego, CA, USA). Pairwise comparisons were made with non-parametric Mann- Whitney U tests with p-value ≤ 0.05 considered significant. In experiments with more than one experimental variable, statistical comparisons were made with two-way ANOVA using Tukey’s multiple comparisons test. P value greater than 0.05 was considered non-significant.

### NanoString analysis

NanoString analysis was performed using conjunctival RNA from female C57BL/6 J mice subjected to 5 days in one of three experimental conditions: unstressed with daily gavage of PBS, DS with daily gavage of PBS, or DS with daily gavage of tributyrin (n = 4 mice per group). Two hundred fifty-four target transcripts were quantified with the NanoString nCounter multiplexed platform using the Mouse Inflammation V2 panel (www.nanostring.com). nCounts of mRNA transcripts were normalized using the geometric means of 6 reference genes (*Cltc*, *Gapdh*, *Gusb*, *Hprt*, *Pgk1*, *Tubb5*). Data was analyzed by ROSALIND® (https://rosalind.onramp.bio/), with a HyperScale architecture developed by ROSALIND, Inc. (San Diego, CA) as described previously^35^. Heatmaps were constructed using GraphPad Prism 9.0 software (GraphPad Software Inc., San Diego, CA, USA).

## Results

### C57BL/6 J mice express the SCFA transporter SLC5A8 on the ocular surface

**Figure 1:**
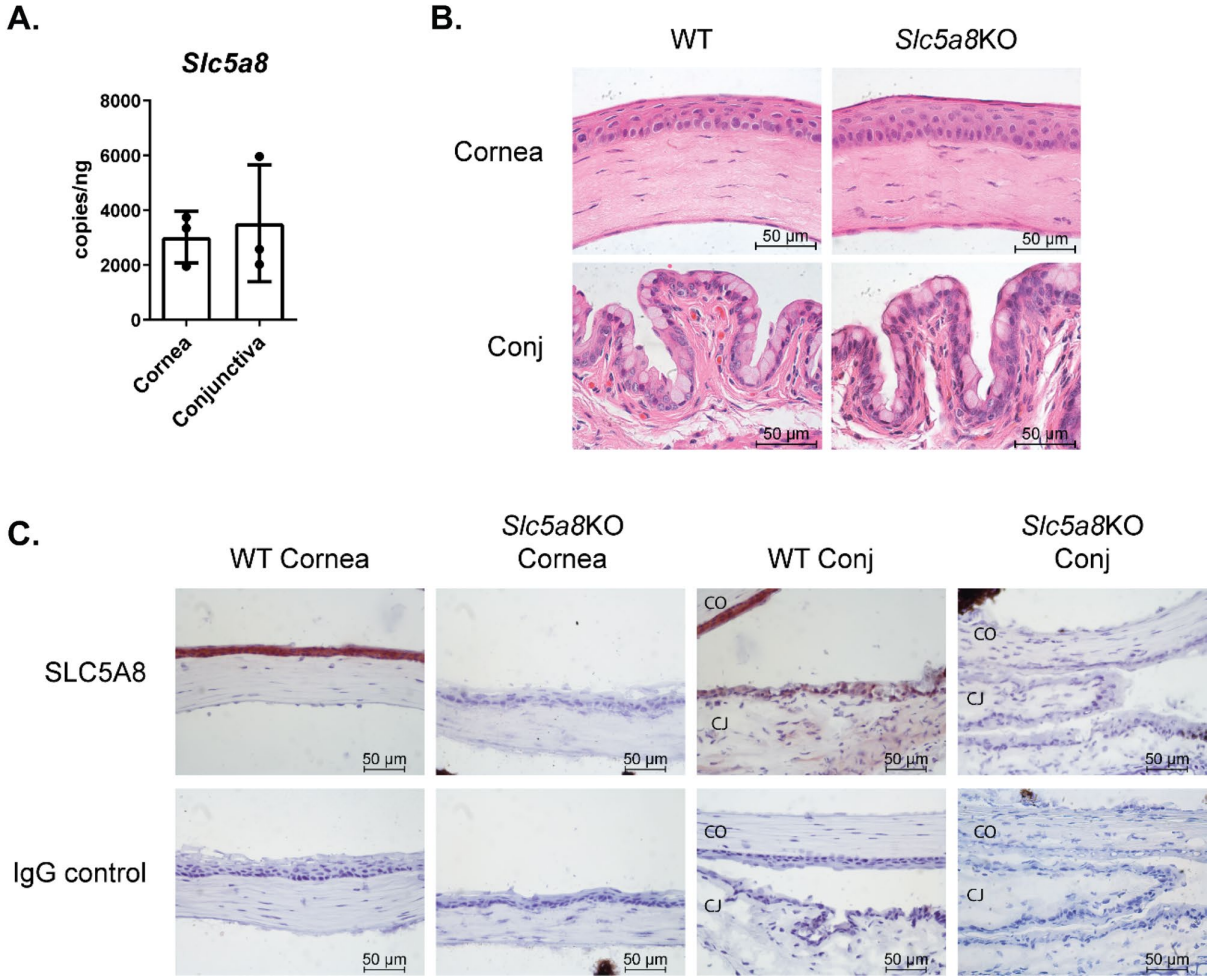
C57BL/6 J mice express mRNA and protein for the SCFA transporter SLC5A8 on the ocular surface. (**A**) Quantitative digital PCR was used for amplification of cDNA prepared from wild-type C57BL/6 murine corneal epithelium and conjunctival tissue. Each datapoint represents tissue from one animal. The number of transcripts detected was normalized to the concentration of DNA. (**B**) Cornea and conjunctival (Conj) sections from wild-type (WT) C57BL/6 and Slc5a8 knockout (KO) mice were stained with H&E to assess morphology. Representative images are shown. (**C**) Cornea and conjunctival (Conj) sections from wild-type (WT) C57BL/6 and *Slc5a8* knockout mice were immunostained with anti-mouse SLC5A8 antibody using chromogenic detection (reddish-brown). Nuclei were stained with hematoxylin (purple). Slides stained with anti-rabbit IgG are shown as a negative control. Representative images are shown. CO = cornea; CJ = conjunctiva.

Originally identified in renal tissue, the monocarboxylate transporter SLC5A8 is widely expressed in many tissues and has been studied extensively in the intestine where its protein is located in the apical lumen-facing membrane in direct contact with SCFAs produced by the intestinal microbiota^36^. We examined whether SCFAs could act directly on ocular surface tissue by testing for expression of transporter mRNA in corneal epithelium and conjunctival tissue in the mouse using quantitative digital PCR. *Slc5a8* transcripts were detected in both corneal epithelium and conjunctiva (Fig. 1A). We next looked for expression of the transporter protein in corneal and conjunctival epithelium. Tissue sections encompassing the eye and ocular adnexa, including conjunctival tissue, were stained with H&E to assess the overall morphology of cornea and conjunctiva in *Slc5a8* KO mice as compared to wild-type (Fig. 1B). The structure and integrity of the corneal epithelium in *Slc5a8* KO mice matched that of wild-type mice. To examine protein expression of *Slc5a8*, separate tissue sections were immunostained and chromogenic detection for SLC5A8 was performed (Fig. 1C). Robust protein expression was detected for the SLC5A8 transporter throughout the corneal epithelial surface and conjunctival epithelium surrounding the goblet cells, with highest expression in the corneal epithelium. As expected, no SLC5A8 immunoreactivity was observed in the same tissue from knockout mice lacking the SLC5A8 protein. The presence of SLC5A8 suggests that SCFAs, in particular butyrate, may interact directly with ocular barrier tissues.

### Butyrate suppresses LPS-induced inflammation in vitro in corneal epithelium and dendritic cells

**Figure 2:**
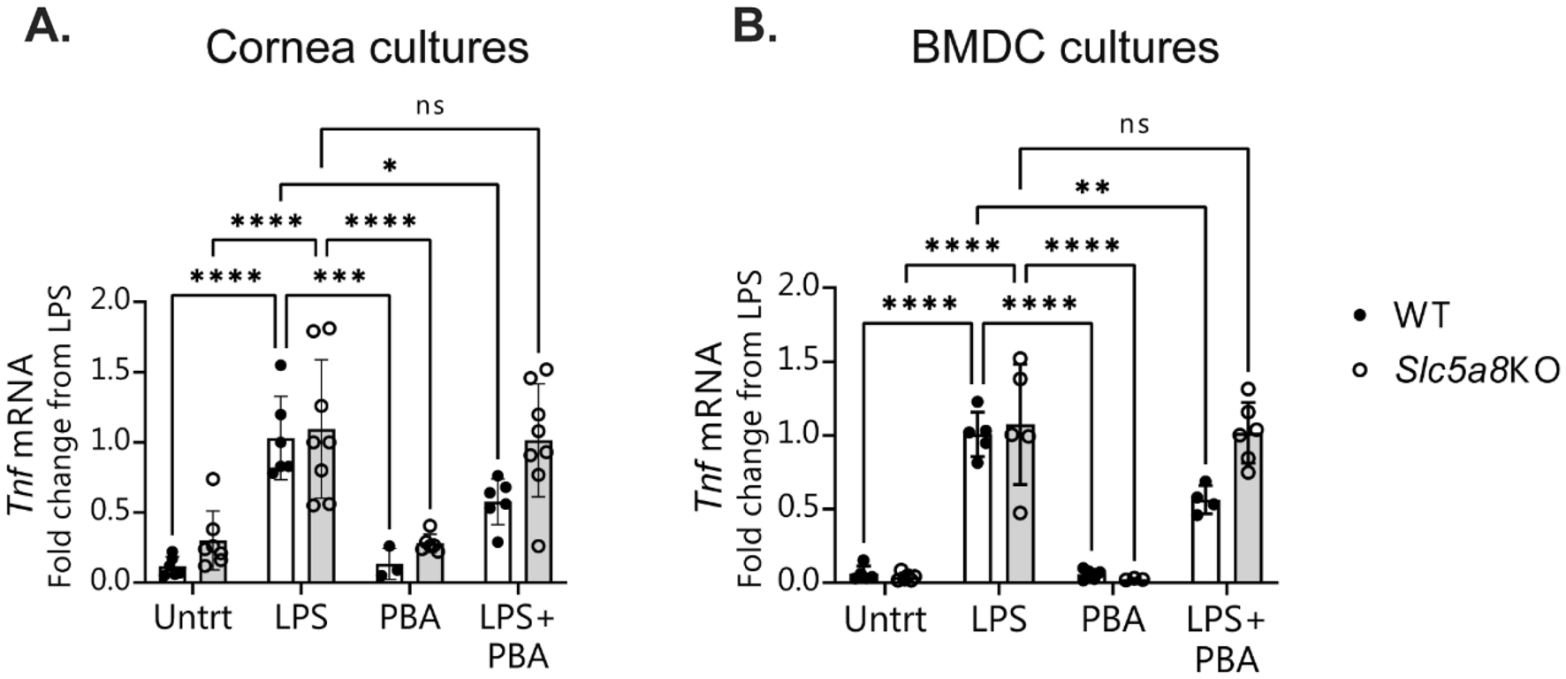
Phenylbutyrate (PBA) treatment of corneal epithelial cells and bone-marrow-derived dendritic cells inhibits LPS-induced inflammation in vitro and the butyrate transporter SLC5A8 is required for this effect. (**A)** Corneal epithelial cells and (**B**) bone marrow dendritic cells (BMDC) were cultured from wild-type (WT) and *Slc5a8* knockout (KO) mice, pretreated with either phenylbutyrate (PBA) or media control for 1 h, then left untreated (Untrt) or challenged with LPS. Each data point is a well with 1 tissue explant, and each condition was in duplicate or triplicate wells within each experiment. Shown is the combined qPCR data from the experiments after each dataset was normalized to the LPS-only treated condition. Mean ± standard deviation of three independent experiments; each experiment used 2–3 wells per condition. Statistical comparisons were made with two-way ANOVA using Tukey’s multiple comparisons test. *P* value greater than 0.05 was considered non-significant (ns). *P ≤ 0.5, **P ≤ 0.01, ***P ≤ 0.001, ****P ≤ 0.0001.

Butyrate has been demonstrated to counteract inflammatory stimuli in colonic epithelial cells^37–41^. We investigated the potential for butyrate to similarly blunt inflammation at the ocular surface in corneal epithelium. Lipopolysaccharide (LPS) has been shown previously to induce pro-inflammatory cytokine expression at the ocular surface in vivo when topically applied to mouse corneas and in vitro when added to corneal and conjunctival epithelial cultures^4,34^. We cultured murine corneal explants in vitro and pre-incubated them with phenylbutyric acid (PBA) before exposure to LPS. PBA is an aromatic short-chain fatty acid which is a chemical derivative of butyric acid, and like butyrate, it also has HDAC activity. PBA is FDA-approved for use in humans and is, therefore, an attractive candidate for possible treatment of ocular surface inflammation. As expected, induction of the inflammatory cytokine *Tnf* was observed in the corneal epithelial cultures after exposure to LPS. Pretreatment of cultures with PBA significantly reduced the induction of *Tnf* mRNA expression (Fig. 2A). No statistical difference was seen in the reduction of the inflammatory response between corneal cells pretreated with either 0.5 mM, 1 mM, or 5 mM PBA (data not shown). Treatment of cultures with PBA without LPS stimulation did not change *Tnf* expression from that of untreated control cultures.

Activated dendritic cells also play an important role in the inflammatory response at the ocular surface^2^. We examined the effects of butyrate treatment on the inflammatory response of bone-marrow-derived dendritic cells (BMDCs) that had been exposed to LPS. Similar to corneal epithelial cells, BMDCs responded to pretreatment with 0.5 mM PBA with a twofold reduction in the expression of *Tnf* after LPS exposure (Fig. 2B).

### Slc5a8 is required for the protective effect of butyrate in cornea and dendritic cells in vitro

The SCFA transporter *Slc5a8* is critical for the anti-inflammatory effects of butyrate in the colon and for intestinal mucosal immune tolerance mediated by dendritic cells exposed to butyrate^40,42^. We investigated whether *Slc5a8* was required for the protective effect of PBA in corneal epithelium and BMDCs. Corneal explants and BMDCs from *Slc5a8* knockout mice were cultured and exposed to LPS in the presence or absence of PBA. In both cell types, PBA pretreatment was unable to reduce *Tnf* mRNA expression in the absence of *Slc5a8* (Fig. 2A and B). This suggests that the transporter is required for butyrate to exert its protective effects against inflammatory stimuli.

### Administration of tributyrin (TB) to mice undergoing desiccating stress protects against corneal barrier disruption and loss of conjunctival goblet cells.

**Figure 3:**
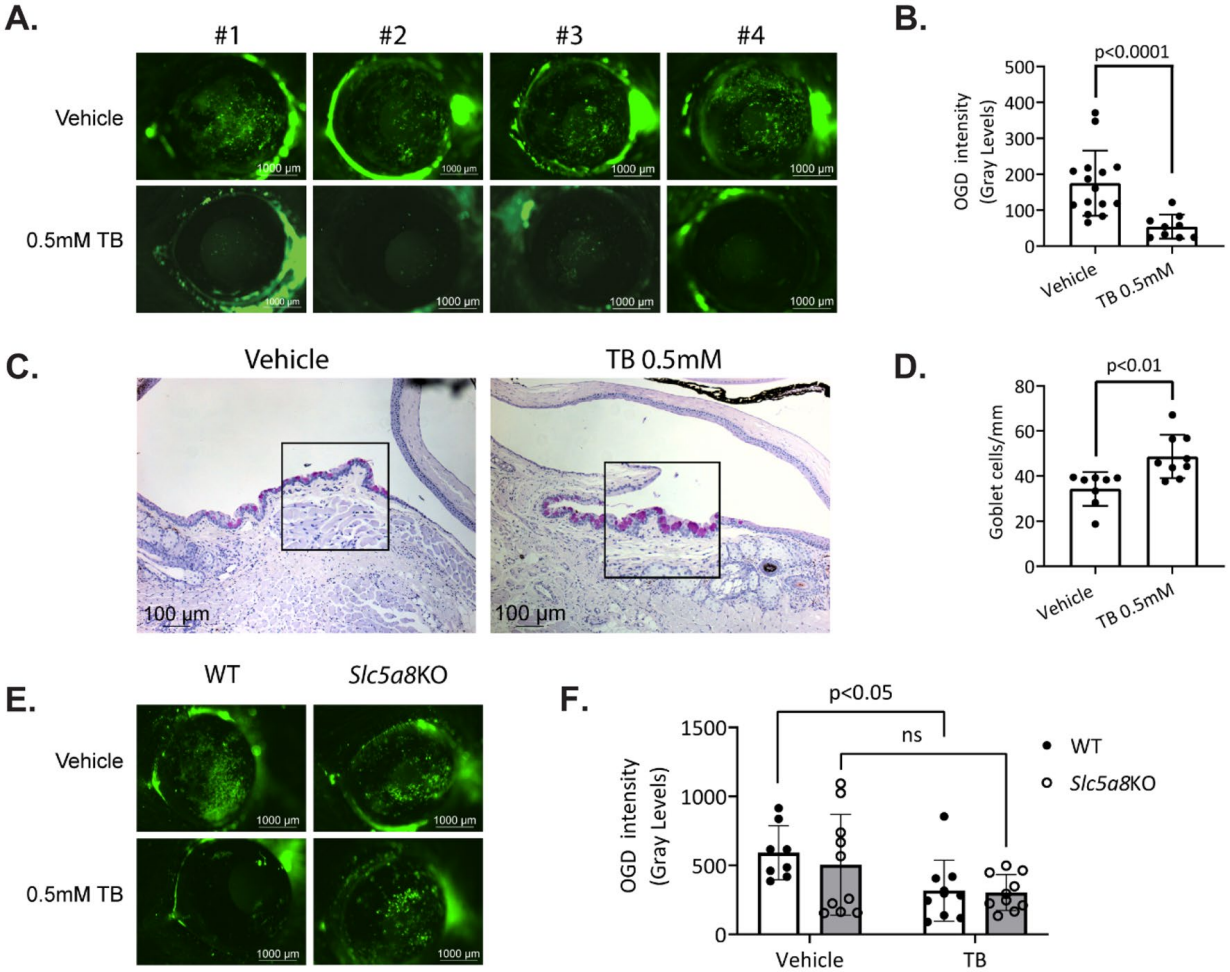
Administration of tributyrin (TB) to mice undergoing desiccating stress protects against corneal barrier disruption and loss of conjunctival goblet cells. (**A**) Representative images of corneal permeability to Oregon-green-dextran (OGD) of four different animals from each treatment group. (**B**) Corneal epithelium permeability to OGD was quantified by measuring the mean fluorescence intensity. Each data point represents the average value from both eyes of one animal, n = 8–15 animals. (**C**) Representative images of conjunctival cryosections with PAS-stained goblet cells (purple-magenta) from TB and vehicle control-treated (PBS) mice. Area demarcated by the square is high magnification of the area underneath. (**D**) Accumulative data for conjunctival goblet cell number per mm, n = 8–9. (**E**) Representative images of corneal permeability to Oregon-green-dextran (OGD) of animals from each treatment group. (**F**) Corneal epithelium permeability to OGD for wild-type (WT) and *Slc5a8* knockout (KO) mice gavaged with either vehicle control or tributyrin. Statistical comparisons were made using two-way ANOVA using Tukey’s multiple comparisons test. P value greater than 0.05 was considered non-significant (ns).

We investigated whether butyrate could exert anti-inflammatory effects at the ocular surface in vivo via administration through the gut using tributyrin. Tributyrin is a stable and rapidly absorbed prodrug form of butyric acid found naturally in butter; upon oral ingestion, tributyrin is hydrolyzed by pancreatic and gastric lipases into glycerol and three butyrate molecules^43,44^. Tributyrin has “generally recognized as safe” (GRAS) classification from the Food and Drug Administration (FDA: 21CFR184.1903) and is used as a food additive by many industries; as such it is a potential candidate for treatment of ocular surface inflammation. In the desiccating stress (DS) model for dry eye, mice are subjected to a low humidity environment and cholinergic blockade which results in compromised corneal barrier function, reduced goblet cell numbers in the conjunctiva, and upregulation of ﻿innate inflammatory pathways^45–47^. Because dry eye is more frequent in women^48,49^, and male mice are resistant to corneal barrier disruption (a hallmark of dry eye)^21,50^, only female mice were used. Female C57BL/6 J mice were subjected to desiccating stress for five days with concurrent daily gavage of either tributyrin or vehicle. After five days of DS, mice were sacrificed and assessed for corneal barrier disruption and goblet cell loss. Corneal permeability was examined using Oregon-Green-Dextran dye (OGD) applied to the corneal surface. Corneas from mice gavaged with 0.5 mM tributyrin during desiccating stress exhibited significantly less permeability to OGD compared to corneas from mice gavaged with vehicle control. This was quantified by measuring the mean fluorescence intensity within a 2 mm circle placed on a digital image of the cornea (Fig. 3A and B).

Another consequence of DS in the murine eye is the loss of goblet cells within the conjunctival epithelium, which normally secrete mucins that form the inner layer of the tear film. After desiccating stress for 5 days, mice orally treated with tributyrin maintained larger densities of goblet cells in the conjunctiva compared to mice treated with vehicle, and goblet cell size appeared larger and more regular (Fig. 3C and D). This suggests that butyrate metabolized in the gut can exert protective effects at the ocular surface in vivo.

We next investigated whether the SLC5A8 transporter is required for the protective effect of butyrate in vivo by administering tributyrin to *Slc5a8* knockout mice concurrently alongside wild-type mice in the desiccating stress environment. Unlike the wild-type cohort, *Slc5a8* knockout mice receiving tributyrin did not show statistically significant improvement in corneal barrier integrity compared to mice receiving vehicle control (Fig. 3E and F), supporting the in vitro results. However, while not statistically significant, the average OGD intensity for tributyrin-treated knockout mice was lower than for vehicle-treated mice, indicating that in vivo butyrate may exert its protective effects only partially through the SLC5A8 transporter.

In intestinal epithelial cells, expression of the *Slc5a8* gene has been shown to be upregulated by probiotic *Lactobacillus plantarum*^51^ and downregulated during inflammation and colon cancer^36^. Germ-free mice have significantly lower *Slc5a8* expression levels in the intestine that are restored by recolonization with bacteria, suggesting that commensal bacteria stimulates expression^52^. To determine if expression of *Slc5a8* changes in the corneal epithelium during DS or after tributyrin treatment, we performed qPCR on corneal epithelium isolated from mice. No significant difference was observed either in response to DS or in response to tributyrin treatment under DS conditions (data not shown).

### Oral tributyrin decreases type I interferon signaling pathway genes in the conjunctiva

**Figure 4:**
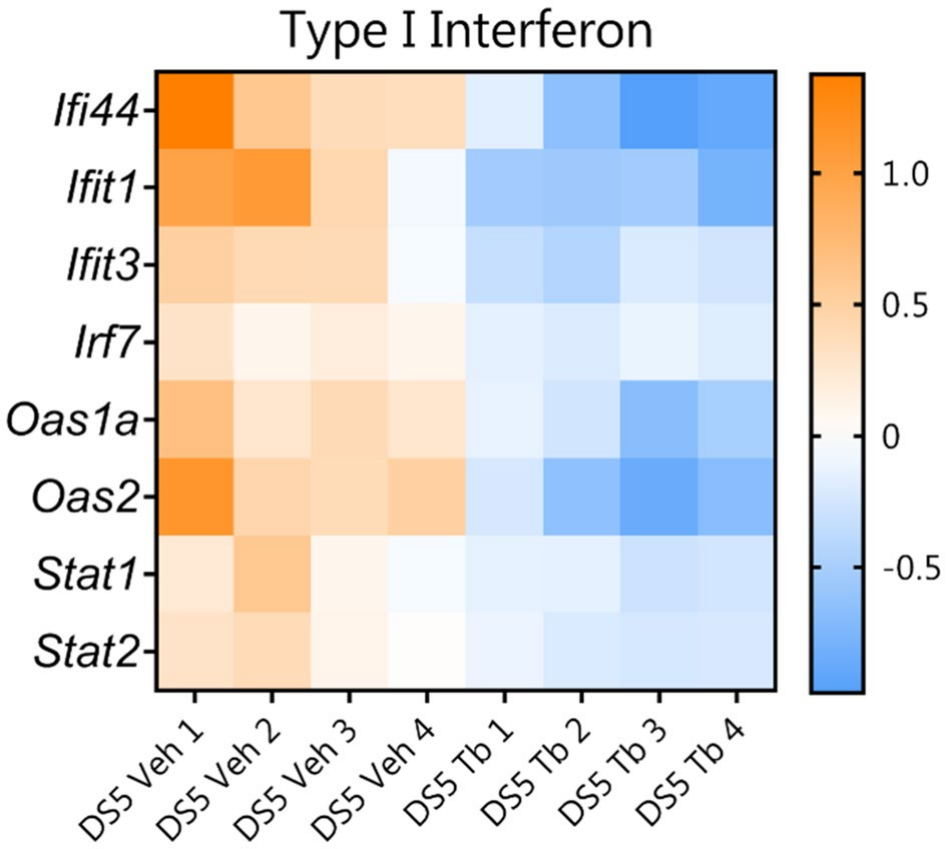
Heatmaps showing that oral tributyrin treatment (Tb) reduces expression of Type I interferon (IFN) signaling genes in conjunctiva. Expression analysis using the NanoString Mouse Inflammation panel v2 was performed on conjunctival tissue from mice subjected to DS with vehicle (Veh) or with tributyrin treatment. For all genes identified as significantly changed, log2 > 1.2 and *p *adj. < 0.05.

To gain insight into the mechanism for butyrate’s protective effects on the ocular surface, we performed gene expression analysis using NanoString on conjunctival tissue isolated from mice subjected to DS with or without tributyrin treatment (n = 4 mice per group). The NanoString Inflammatory panel allows for interrogation of 248 genes involved in the inflammatory response and related pathways (www.nanostring.com). Eight genes were significantly changed, all of which were downregulated by tributyrin treatment (*Ifi44*, *Ifit1*, *Ifit3*, *Stat2*, *Oas2*, *Oas1a*, *Stat1*, *Stat2*; log2 fold change > 1.2, p-adj < 0.05, Fig. 4). All of these genes are involved in Type I interferon (IFN) signaling^53,54^. Type I IFNs have several functions, including activating intracellular antimicrobial responses and modulation of innate and adaptive immune responses^53^. Butyrate has been shown in other studies to modulate genes involved in Type I IFN signaling^55,56^.

## Discussion

The gut microbiota plays many important roles in host health, including in host nutrition, physiology, metabolism, and pathogen resistance. One important function of the commensal gut bacteria is its ability to modulate inflammatory responses. Several studies have shown that a healthy gut microbiota promotes ocular homeostasis and disease resistance. Germ-free mice are more susceptible to ocular *Pseudomonas aeruginosa* infection due to deficiency of a normal ocular surface immune response, which can be reversed by recolonizing the gut with bacteria^19^. Our group has shown that the presence of an intact health microbiota protects against keratoconjunctivitis sicca in several different dry eye disease models^20–22^. Conversely, it has been shown that a cross-reactive antigen from a gut commensal primes autoreactive retina-specific T-cells and triggers disease in a murine ﻿autoimmune uveitis model^5^. Our study adds to a growing body of knowledge regarding ocular inflammation and the relationship between the eye and the gut microbiota.

In this study, we show that the SCFA transporter *Slc5a8* is expressed at the ocular surface in conjunctival and corneal epithelium both at mRNA and protein level. This suggests that circulating butyrate may directly interact with the ocular surface. The precise mechanisms behind the interaction between the gut and the ocular surface are not yet clear; the bacteria-produced small metabolite butyrate is one potential mediator as it has been shown to modulate inflammatory responses both in the gut and elsewhere in the body^12,23–29^. SCFAs acetate, propionate, and butyrate are produced by bacteria through fermentation of non-digestible dietary fiber in the large intestine, and the colonic epithelium expresses several proteins that either bind these molecules as part of signaling cascades or transport them across the cellular membrane. Butyrate in the gut has been shown to enter the bloodstream; elevated butyrate levels in serum can be measured after oral administration of butyrate and tributyrin^43,44,57–59^. We hypothesize that butyrate originating in the gut could be transported to the ocular surface via the vascular system and directly interact with cells at the ocular surface.

We show that tributyrin treatment helps to restore corneal barrier integrity and goblet cell loss that occurs under DS conditions. It has been previously observed that germ-free mice, which are devoid of microbes including commensals that produce SCFAs in the gut, have lower goblet cell density not only in the gut but also in the eye^60,61^. It is known that in the intestinal epithelium butyrate helps to maintain the intestinal barrier by modulating goblet cell expression of mucins and goblet cell differentiation^62,63^. It is possible that butyrate produced in the gut also has a direct effect on the differentiation or maintenance of conjunctival goblet cells.

Butyrate also functions as a ligand for a subset of G protein-coupled receptors that bind SCFAs, including FFAR2 and HCAR2, which are expressed in epithelial barrier surfaces including the gut. There is a large body of work on the function of these receptors in the colonic epithelium and immune cells^28,64,65^. In the eye, HCAR2 signaling is essential for the ability of hydroxybutyric acid to inhibit the expression of pro-inflammatory markers in the retinal epithelium^66^. Our in vivo results with tributyrin-fed *Slc5a8* knockout mice indicate that SLC5A8 may be only partially required for tributyrin’s protective effect on corneal barrier function. It will be important to address the potential role of butyrate signaling via HCAR2 and FFAR2 in future studies.

We also show that the direct application of phenylbutyric acid (PBA) can reduce the inflammatory response in corneal epithelial cells. Corneal cultures pretreated with PBA expressed lower levels of inflammatory cytokines when exposed to LPS, supporting the hypothesis that butyrate can directly reduce inflammation at the ocular surface. Interestingly, PBA’s effect is lost in the absence of SCFA transporter SLC5A8, suggesting that butyrate is transported into the cell. Butyrate can directly modulate gene expression in the cell by inhibiting histone deacetylases (HDACs)^12,67–69^. It is possible that butyrate modulates the expression of inflammatory markers in corneal epithelial cells by HDAC inhibition. In support of this, in vitro studies with a corneal epithelial cell line showed that activation of toll-like receptor 3 causes barrier disruption by increasing HDAC1 which in turn represses the transcription of E-cadherin; this barrier disruption can be blocked with butyrate^70^. It would be very interesting to test SCFAs propionate and acetate in future studies with our model, particularly since propionate, but not acetate, also has HDAC inhibitory properties and can be transported into the cell by SLC5A8^71^. We will investigate the potential role of butyrate’s HDAC inhibitory activity in suppressing the inflammatory response in corneal epithelium in future studies.

Our results indicate that both epithelial cells and antigen presenting cells responded to butyrate by decreasing inflammatory *Tnf* mRNA. We have previously shown that both corneal epithelium and BMDCs respond to LPS by secreting inflammatory cytokines^34^. Our study adds to the literature showing another bacteria by-product has an anti-inflammatory effect on the ocular surface and antigen-presenting cells (APCs). It is unknown at the moment if this effect happens simultaneously or if the effect on epithelial cells follows the effect on APCs. Further testing will be needed to dissect this question.

We and others have previously shown that Treg cells are protective at the ocular surface^22,46^ and that desiccating stress and age-related ocular surface disease induce dysfunctional regulatory T cells^72,73^. Butyrate produced in the gut has also been shown to impact inflammation indirectly at distal sites by increasing the levels of peripheral anti-inflammatory Tregs^11,23,24,27^. The addition of butyrate to naive CD4^+^ T cells cultured in Treg cell-polarizing conditions results in increased histone acetylation at the Foxp3 promoter, implicating the HDAC inhibitory activity of butyrate as a mechanism for increased Foxp3 expression and thus Treg differentiation^11^. In fact, it has been shown that spleen-derived dendritic cells treated with butyrate induce differentiation of naïve T cells into FoxP3^+^ Treg cells and suppress differentiation into pro-inflammatory interferon-γ + T cells, and the SLC5A8 transporter is required^42^. Oral administration of sodium butyrate (NaB) in the experimental autoimmune uveitis (EAU) mouse model increased the ratio of anti-inflammatory Treg cells to Th17 cells, resulting in less disease^27^. Interestingly, another study using the EAU model showed that propionate, another SCFA with HDAC inhibitory activity, can also induce Tregs and suppress T effector cell induction, resulting in less disease^26^. It is possible that facilitation of Treg differentiation over pro-inflammatory T-cells by butyrate may also play a role in butyrate’s protective effect at the ocular surface. Based on all these previous findings, we would predict that mice treated with tributyrin have increased numbers of FoxP3^+^ Tregs at the ocular surface and will further investigate this in follow-up studies.

Finally, we show that in the conjunctiva of mice subjected to desiccating stress, there is upregulation of genes involved in Type I IFN signaling, and tributyrin treatment reduces expression of these genes. This result agrees with our previously published NanoString results comparing gene expression in human conjunctival cells from Sjögren Syndrome (SS) patients with dry eye and healthy control subjects, where we observed upregulation of Type 1 IFN signaling in SS^35^. SS is an autoimmune inflammatory disorder characterized by secretory dysfunction in the eye and mouth; in the eye, this results in tear film instability, reduced tear production, and corneal barrier disruption. Increased expression of type I IFNs genes has been shown in autoimmune disorders including SS, and type I IFNs have been implicated in lacrimal gland and salivary gland pathology in human SS and in mouse models of SS^74–76^. In addition, tears isolated from mice undergoing DS have elevated IFN-*α*^77^. There is evidence in the literature that microbial products including butyrate can modulate Type I IFN signaling^55,56^. Butyrate may be acting directly at the ocular surface to downregulate inflammatory Type I IFN signaling, possibly via its HDAC inhibitory activity to modulate Type I IFN gene expression.

**Figure 5:**
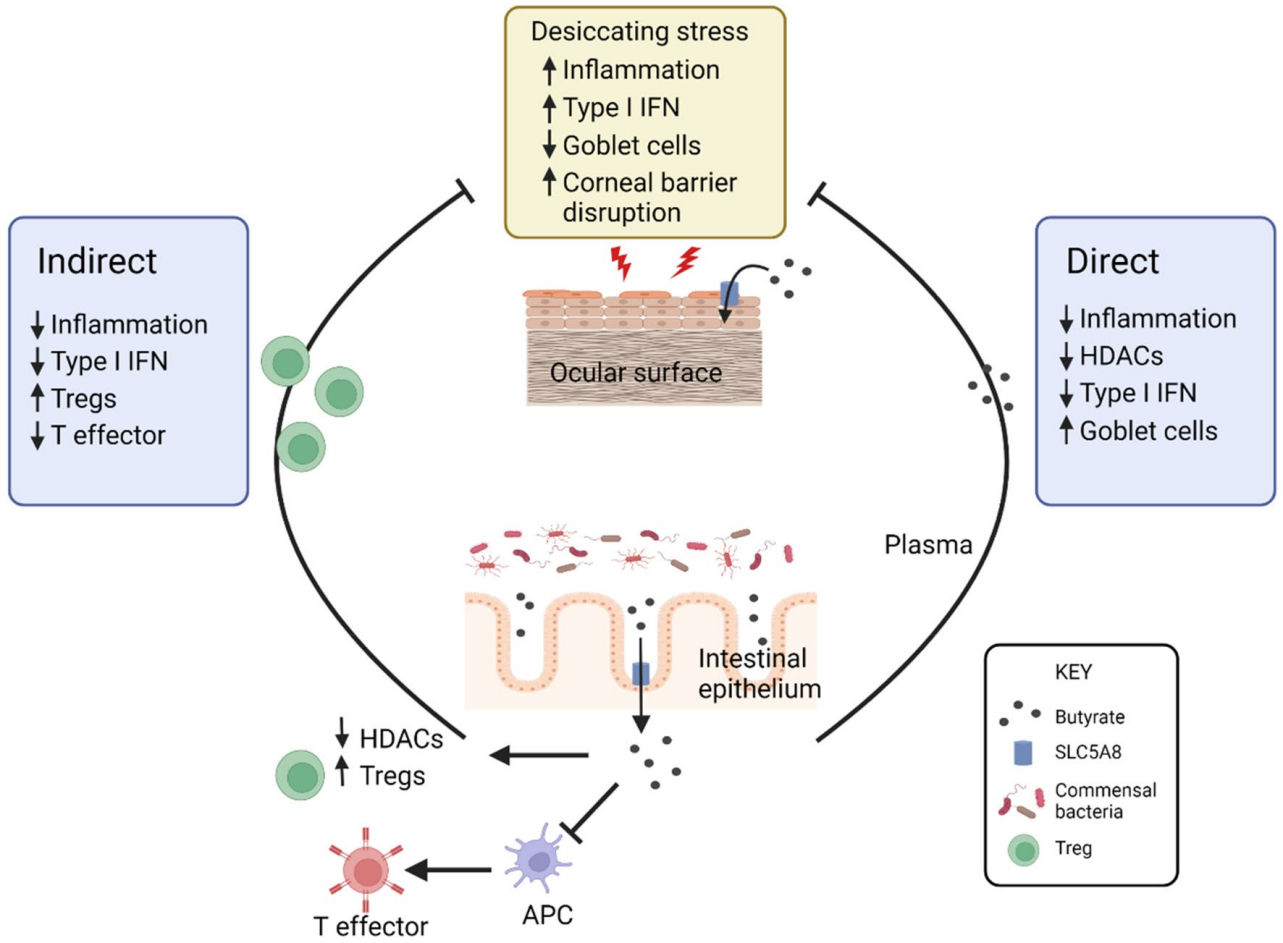
Proposed mechanisms for butyrate’s protective effect on the ocular surface. Butyrate may impact the ocular surface in two different ways, indirectly though modulation of immune cells and directly through interaction with ocular surface epithelium expressing SLC5A8 or other SCFA receptors and modulating gene expression through its HDAC inhibitory activity. APC, Antigen presenting cell; HDAC, histone deacetylase; IFN, interferon; Treg, regulatory T cell. Figure drawing created with BioRender.com.

Our data show that butyrate originating in the gut can modulate inflammatory responses at the ocular surface. We propose two possible mechanisms for butyrate’s action on the ocular surface, an indirect mechanism via modulation of immune cells and a direct mechanism in which butyrate interacts directly with the ocular surface, based on our findings and on other data in the literature (Fig. 5). As discussed, there is considerable support in the literature for both scenarios. In this study, expression of SCFA receptors and the SCFA transporter SLC5A8 at the ocular surface suggest that circulating butyrate can interact directly with ocular surface epithelium. In support of this, butyrate suppressed pro-inflammatory gene expression in corneal explants in vitro and required the SLC5A8 transporter for this effect. Finally, butyrate delivered intragastrically ameliorates ocular surface disease in a dry eye disease mouse model. Together these findings contribute to a growing body of knowledge on the links between the gut microbiota and the health of the eye.

## Data Availability

The NanoString data for this study can be found in the GEO repository (Submission ID GSE195578, https://www.ncbi.nlm.nih.gov/geo/).

## Abbreviations

APC: Antigen presenting cell
BMDC: Bone marrow-derived dendritic cells
DS: Desiccating stress
HDAC: Histone deacetylase
KO: Knock-out
LPS: Lipopolysaccharide
OGD: Oregon green dextran
MFI: Mean fluorescence intensity
NaB: Sodium butyrate
PBA: Phenylbutyrate
SCFA: Short-chain fatty acid
SS: Sjögren Syndrome
TB: Tributyrin
Treg: Regulatory T cell
WT: Wild-type
