# Author Correction: Gut-derived butyrate suppresses ocular surface inflammation

**DOI:** 10.1038/s41598-022-10856-y

**Published:** 2022-04-21

**Authors:** Laura Schaefer, Humberto Hernandez, Rosalind A. Coats, Zhiyuan Yu, Stephen C. Pflugfelder, Robert A. Britton, Cintia S. de Paiva

**Affiliations:** 1 Center of Metagenomics and Microbiome Research, Department of Molecular Virology and Microbiology, Baylor College of Medicine, 1 Baylor Plaza, Houston, TX 77030 USA; 2 Ocular Surface Center, Department of Ophthalmology, Cullen Eye Institute, Baylor College of Medicine, 6565 Fannin St., NC505, Houston, TX 77030 USA; 3 Present Address: Humberto Hernandez, University of Houston-Victoria, 3007 N. Ben Wilson, Victoria, TX 77901 USA

Correction to: *Scientific Reports* 10.1038/s41598-022-08442-3, published online 16 March 2022

The original version of this Article contained an error in the spelling of the author Cintia S. de Paiva which was incorrectly given as Cintia de Paiva.

In addition, the Author Contributions section now reads:

Conceptualization, L.S., H.H. and C.S.D.P.; Formal analysis, L.S., H.H., R.A.C. and Z.Y.; Funding acquisition, C.S.D.P.; Methodology, L.S., H.H., Z.Y. and C.S.D.P.; Project administration, C.S.D.P.; Supervision, C.S.D.P.; Writing—original draft, L.S.; Writing—review & editing, L.S., H.H., R.A.C., Z.Y., S.C.P., R.A.B. and C.S.D.P.

The original Article has been corrected.

